# Supplementary figures and images for: AT-Hook Transcription Factors Show Functions in Liriodendron chinense under Drought Stress and Somatic Embryogenesis
Source: Plants (Basel). 2023 Mar 17;12(6):1353. doi: 10.3390/plants12061353 (PMC10056439; doi:10.3390/plants12061353)

Tree scale: 1

**Clade I**

**Clade II**

**Clade III**

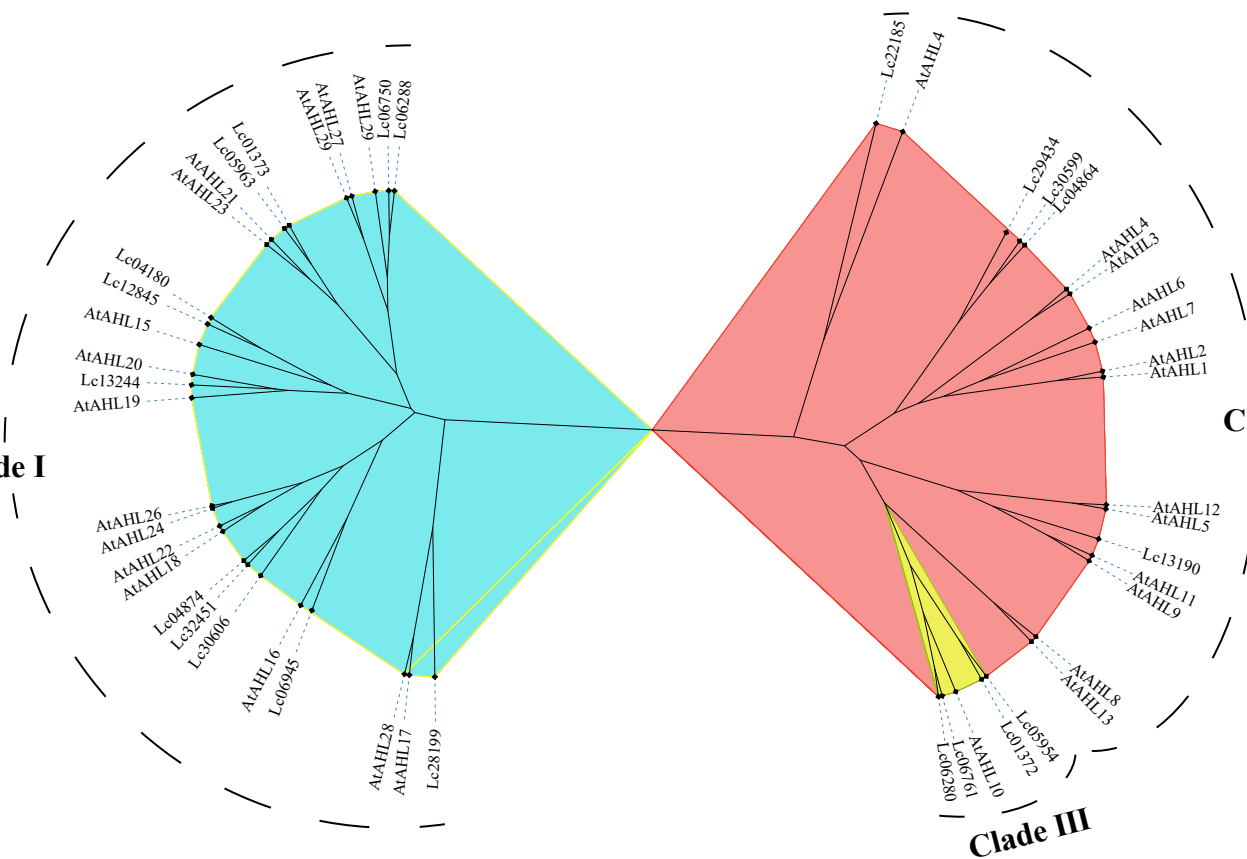

Supplement: Supplementary file 1 [file plants-12-01353-s001.zip › Figure S1 Bayesian tree indicating the phylogenetic relationships between 21 AHL proteins from Liriodendron chinense (Lc) and 29 from Arabidopsis. .pdf]

A

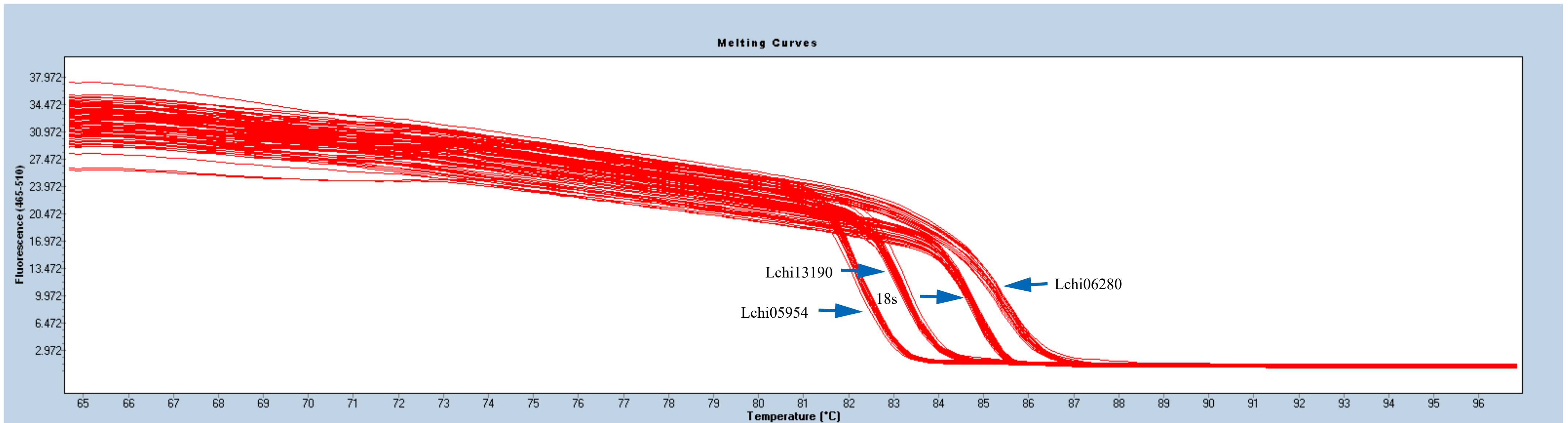

B

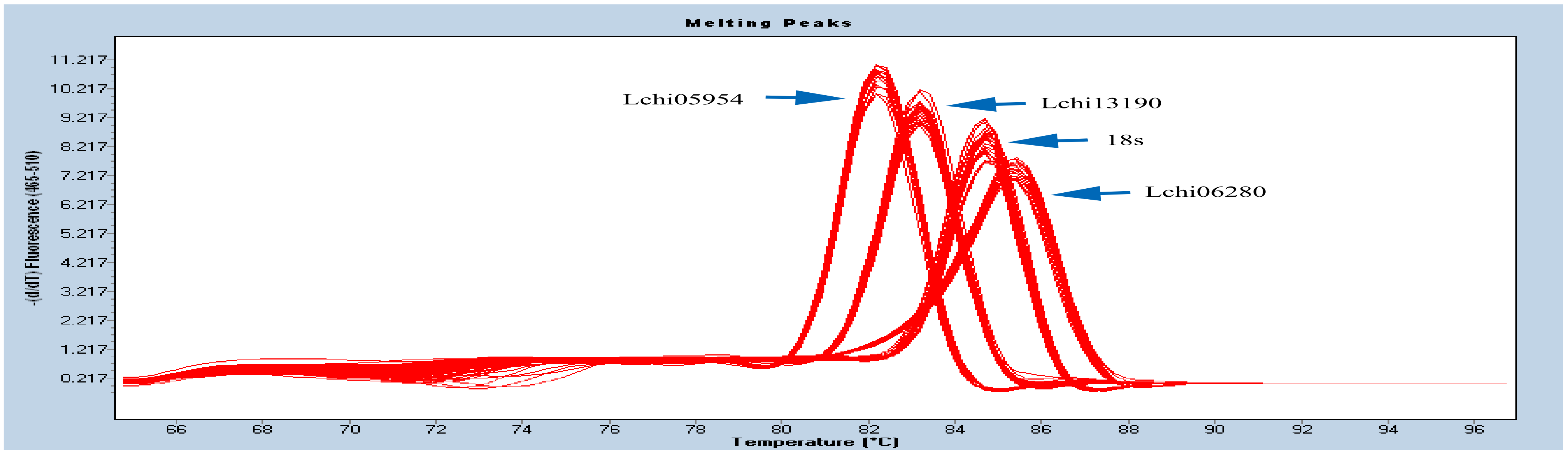

Supplement: Supplementary file 1 [file plants-12-01353-s001.zip › Figure S2 Quantitative real-time PCR (qRT-PCR) dissolution curve and standard curve of three LcAHL genes under drought stress..pdf]

A

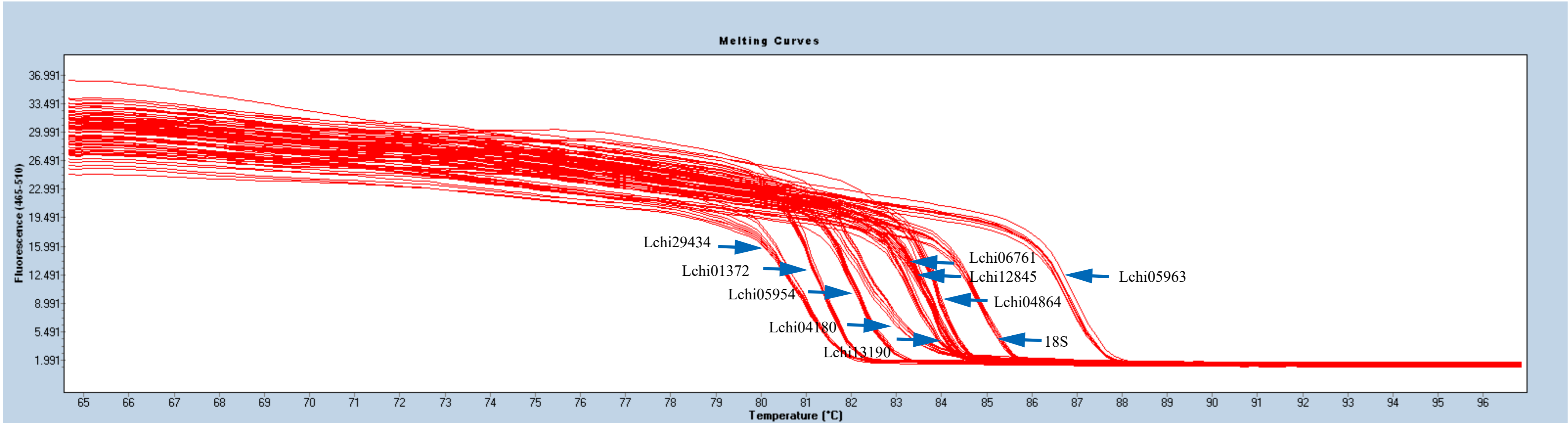

B

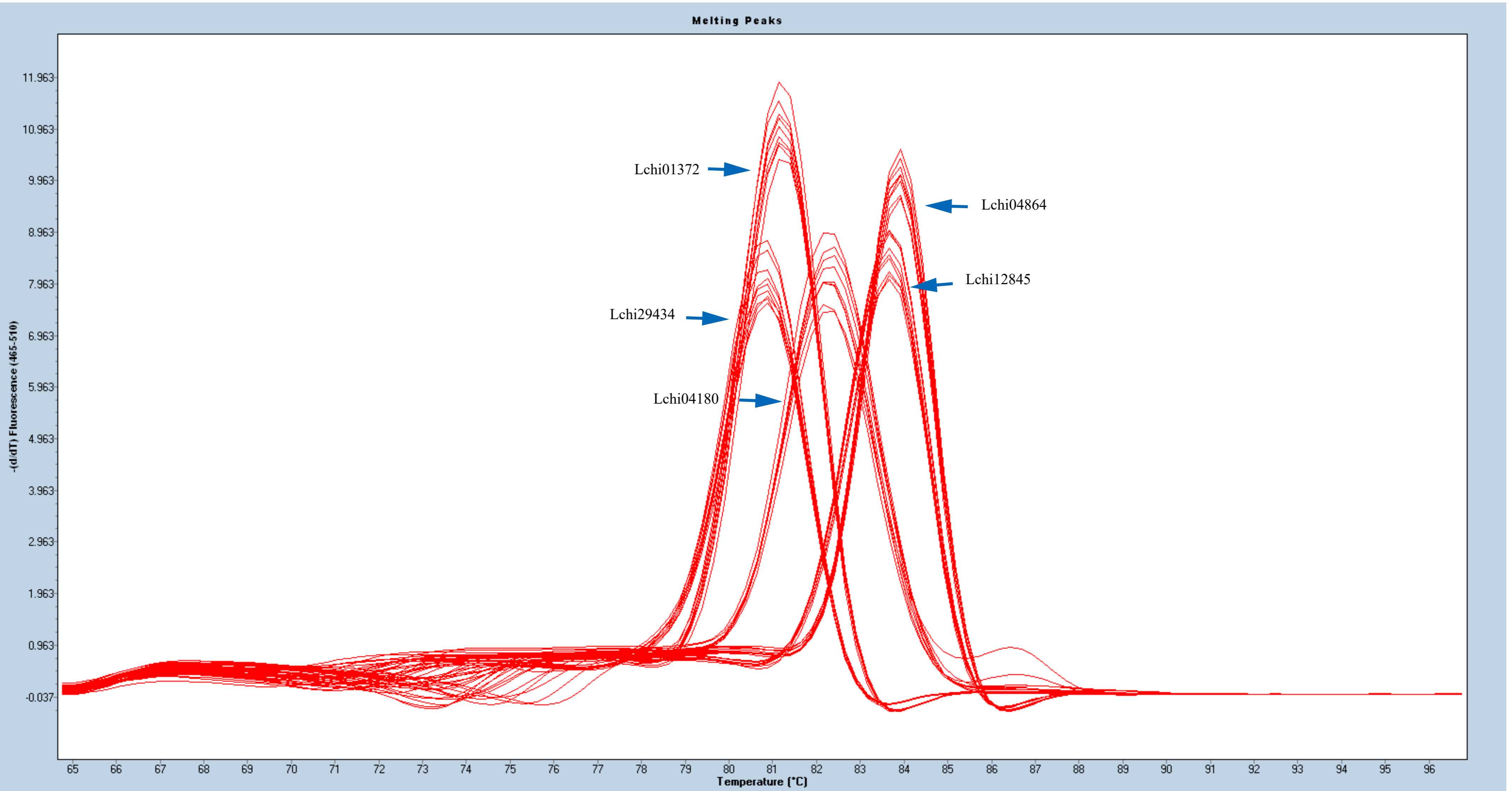

C

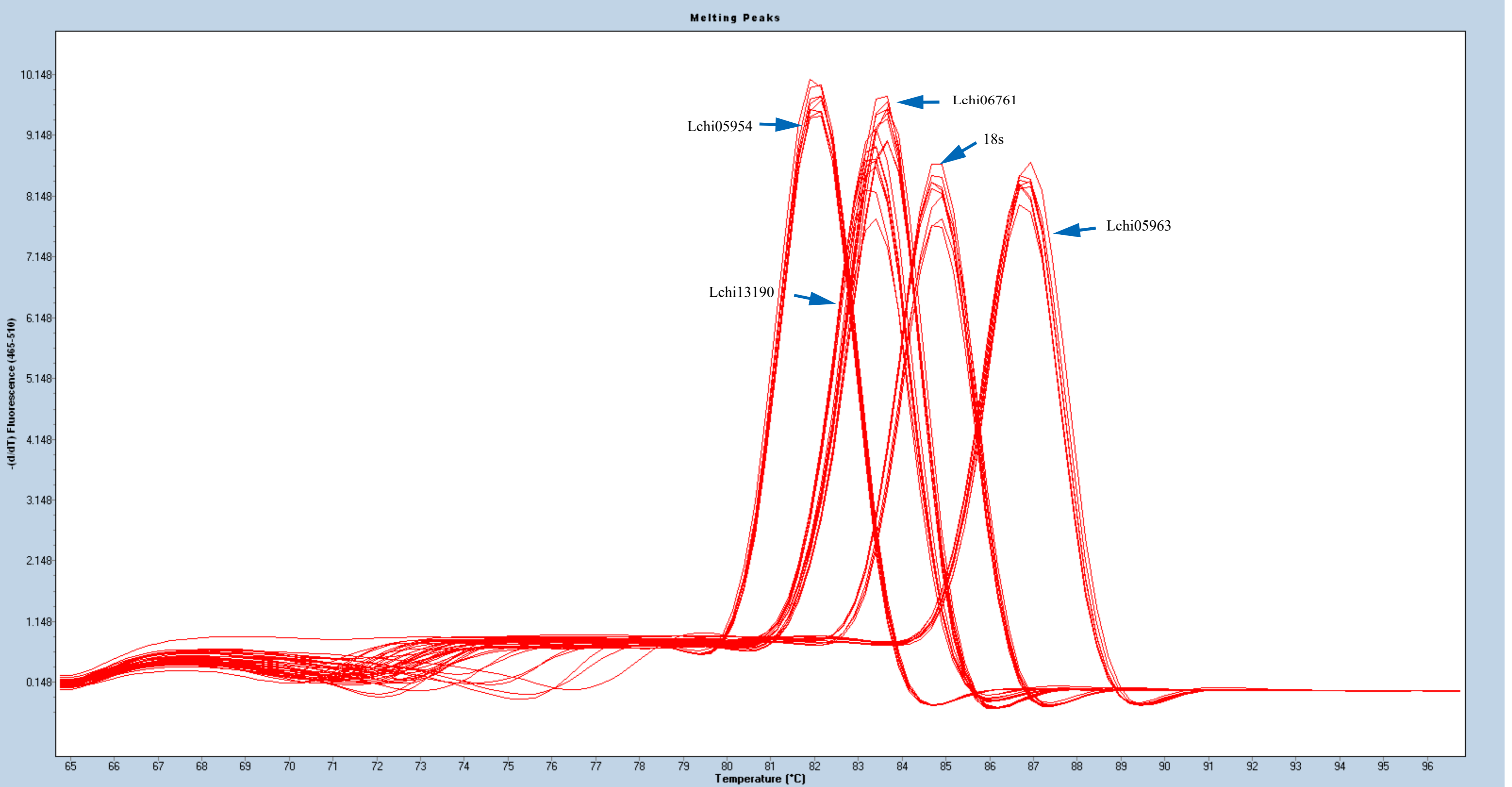

Supplement: Supplementary file 1 [file plants-12-01353-s001.zip › Figure S3 Quantitative real-time PCR (qRT-PCR) dissolution curve and standard curve of nine LcAHL genes at different stages of somatic embryogenesis. .pdf]
